# Transcriptional profiles of WNV neurovirulence in a genetically diverse Collaborative Cross population

**DOI:** 10.1016/j.gdata.2016.10.005

**Published:** 2016-10-14

**Authors:** Richard Green, Courtney Wilkins, Sunil Thomas, Aimee Sekine, Renee C. Ireton, Martin T. Ferris, Duncan M. Hendrick, Kathleen Voss, Fernando Pardo-Manuel de Villena, Ralph S. Baric, Mark T. Heise, Michael Gale

**Affiliations:** a Department of Immunology, and the Center for Innate Immunity and Immune Disease (CIIID), University of Washington, Seattle, WA, USA; b Department of Genetics, University of North Carolina at Chapel Hill, NC, USA; c Department of Epidemiology, University of North Carolina at Chapel Hill, NC, USA

**Keywords:** Collaborative Cross, West Nile virus, Nanostring, Bioinformatics, Flavivirus

## Abstract

West Nile Virus (WNV) is a mosquito-transmitted virus from the Flaviviridae family that causes fever in 1 in 5 infected people. WNV can also become neuro-invasive and cross the blood-brain barrier leading to severe neurological symptoms in a subset of WNV infected individuals [1]. WNV neuro-invasion is believed to be influenced by a number of factors including host genetics. In order to explore these effects and recapitulate the complex immune genetic differences among individuals, we studied gene expression following WNV infection in the Collaborative Cross (CC) model. The CC is a mouse genetics resource composed of > 70 independently bred, octo-parental recombinant inbred mouse lines [2]. To identify the individual host gene expression signatures influencing protection or susceptibility to WNV disease and WNV neuroinvasion, we used the nanostring nsolver platform to quantify gene expression in brain tissue isolated from WNV-infected CC mice at days 4, 7 and 12 post-infection [3]. This nanostring technology provided a high throughput, non-amplification based mRNA quantitation method to detect immune genes involved in neuro-invasion. Data was deposited into the Gene Expression Omnibus (GEO) under accession GSE85999.

**Table:** 

Specifications	
Organism/cell line/tissue	*Mouse, Brain Tissue*
Sex	*Male*
Sequencer or array type	*Nanostring Pan Cancer Immune Panel*
Data format	*Raw and Normalized matrix provided*
Experimental factors	*Infection, control*
Experimental features	*This analysis shows brain specific neurovirulent gene signatures during West Nile Virus infection using the Nanostring platform.*
Consent	*Allowed for reuse. Please contact authors before reuse.*
Sample source location	*Seattle, WA, USA*

## 1 Direct link to deposited data

http://www.ncbi.nlm.nih.gov/geo/query/acc.cgi?acc=GSE85999

## 2 Experimental design

We screened RNA from 95 brain samples across 8 unique CC dRIX (collaborative cross discovery recombinant intercrosses), F1 crosses of the CC recombinant inbred lines (see Table 1). Across the 8 CC dRIX, four were symptomatic and four were asymptomatic following infection. Samples were collected on days 4, 7, and 12 post-WNV infection. The nanostring platform uses the ncounter technology that utilizes 100 nt molecular bar codes (50 nt capture probe and 50 nt reporter probe) which measure gene quantities without an amplification step. We used a predesigned kit (pan cancer immune) that includes 770 immune related genes.

## 3 Material and methods

### 3.1 Virus

West Nile virus TX-2002-HC (WN-TX) was grown using previously described methods [4]. Viral stocks were generated using supernatants collected from infected Vero cell lines and stored at 80 °C.

### 3.2 Mice and infection

CC dRIX lines were bred at the University of North Carolina at Chapel Hill under specific-pathogen-free (SPF) conditions. Male mice were transferred to the University of Washington at 6 to 8 weeks old. Age- and sex-matched 8- to 10-week old mice were subcutaneously inoculated in the rear footpad with 100 PFU WN-TX. Mice were monitored daily for morbidity (percentage of initial weight loss) and clinical disease scores. Mice were housed under BSL-3 conditions throughout the experiments, and tissues were processed under BSL-3 conditions. All animal experiments were approved by the University of Washington Institutional Animal Care and Use Committee. The Office of Laboratory Animal Welfare of the National Institutes of Health (NIH) has approved the University of Washington (A3464-01), and this study was carried out in strict compliance with the Public Health Service (PHS) Policy on Humane Care and Use of Laboratory Animals.

### 3.3 Collaborative Cross dRIX lines and disease definitions

The clinical scoring system used to evaluate WNV-infected mice was as follows: 0, healthy mouse (baseline); 1, ruffled fur, lethargy, hunched posture, no paresis, normal gait; 2, altered gait, limited movement in one hind limb; 3, lack of movement, paresis in one or both hind limbs; 4, moribund. Based on weight loss, clinical scoring, and brain histology, CC dRIXs were segregated into two broad categories: asymptomatic and symptomatic. We evaluated all lines for evidence of WNV within the CNS by qPCR. Symptomatic lines were defined by having weight loss > 10% and/or any death, whereas asymptomatic were defined by having a < 10% weight loss and no death. CNS histopathologic involvement was evaluated by a veterinary pathologist. The CC dRIX featured in this study are listed in Table 1.

### 3.4 RNA extraction and quantitative PCR (qPCR) of WNV

Brain tissue from mock or WNV-infected mice were stored and homogenized in PBS at 5500 RPM using a Precellys 24 machine. Samples were then transferred into tubes containing TRI Reagent. Total RNA was extracted using the Ribopure RNA Purification Kit, with the addition of bromochloropropane. RNA was converted to cDNA using the iScript Select cDNA Synthesis Kit (Biorad). cDNA was assayed for WNV expression through relative expression SYBR Green RT-qPCR, with GAPDH as a loading control.

### 3.5 Nanostring analysis

Nanostring results (raw and normalized counts) were produced from RCC files using the nSolver software (version 2.6). Raw comma delimited files will be exported and uploaded to Rstudio (version 0.99.486) with R (version 3.2.4). Over 95% of the genes on the nanostring panel matched to previous probe matching techniques [8]. Exploratory analysis and summary statistics were calculated to identify variations in the data and relationships among replicates and conditions in each study.

### 3.6 Statistical modeling

**Fig. 2:**
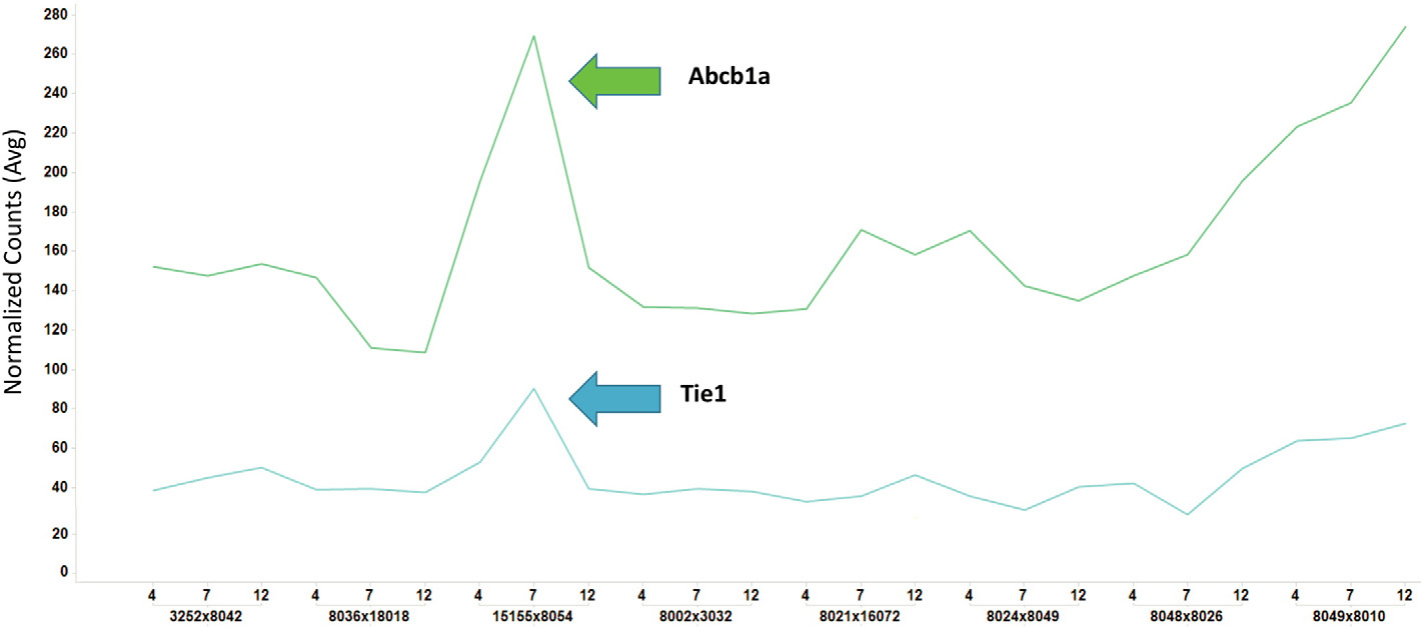
Molecular bar codes show elevation in Abcb1a and Tie1. The gene counts (from the molecular bar codes) were averaged across replicates and normalized to internal housekeeping genes in the nanostring panel. This standard normalization approach was able to identify small increases in Abcb1a and Tie1 within the brain tissue from an asymptomatic line and gradual increases in the symptomatic line.

We assessed the nanostring data using two statistical approaches. In one approach, we normalized the expression data using pre-selected, internal housekeeping genes and plotted subsets of immune related genes in spotfire (Fig. 2). In an alternate approach, we performed differential expression analysis across all genes from filtered gene counts (mean ≥ 20) and then normalized the raw counts using the voom Bioconductor package in limma. Linear modeling was then performed in limma using R [1], [6], [7].

### 3.7 Co-expression

Co-expression was only performed on those genes that were determined to be statistically significant from the differential expression analysis (threshold: log2 fold change ≥ | 0.58 | and FDR ≤ 0.05) in at least one comparison (Mock vs. Infected at days 2, 4, 7 and 12 post-infection). Pearson correlations were run on the union of log2FC using the WGCNA (color modules) and heatmap.2 Bioconductor packages in R [1], [6], [7].

### 3.8 Functional analysis

Ingenuity Pathway Analysis (IPA) and Jepetto (version 1.3.1) were used to determine the biological functions of modules in the co-expression analysis. These tools accept a list of genes and produce a list of known biological functions with an enrichment score corresponding to how significant those genes are to each function.

## 4 Conclusion

Differential expression analysis of the brain tissue from WNV-infected CC dRIX lines identified several hundred (523) up-regulated, statistically significant genes, primarily in CC lines that were symptomatic for disease and qPCR positive for WNV expression in the brain (CC043/GeniUnc_x_CC037/TauUnc, CC016/GeniUnc_x_CC038/GeniUnc, CC061/GeniUnc_x_CC026/GeniUnc, CC038/GeniUnc_x_CC013/GeniUnc). Co-expression analysis revealed large differences in the genetic signatures between those animals that were asymptomatic and those developing neuro-invasive WNV infection. The asymptomatic lines showed strong early activation of innate immune genes associated with the RIG-I-like Receptor (RLR) pathway. Only animals defined as asymptomatic showed an increase in expression at day 4 and day 7 post-infection with WNV. In the asymptomatic lines where WNV was detected in the brain, we found an elevation in genes associated with toll like receptor signaling, antigen presentation, and natural killer cell mediated cytotoxicity at days 7 and 12 post-WNV infection. In the symptomatic animals, the most gene expression occurred at day 12 post-infection, a time point when significant levels of WNV were detected in the brain. The brain tissue isolated from symptomatic animals also demonstrated activation of the innate immune signaling pathways, but unlike the symptomatic lines, the increase in gene activity was sustained throughout the time course, up to day 12 post-infection.

From looking at unique gene activation in CC039/Unc_x_CC020/GeniUnc within the panel, we observed slight increases in genes Abcb1a and Tie1. Abcb1a is part of the family of (ABC) transporters proteins that facilitate the transport molecules across intra and extra cellular membranes. Abcb1a also plays a role in antigen presentation and is involved in the permeability of the blood brain barrier with certain hydrophobic amphipathic drugs. Tie1 (Tyrosine kinase with immunoglobulin-like and EGF-like domains 1) is a gene involved in endothelial cell adhesion and migration through a p38 mechanism [9]. Tie1 was previously reported in immune studies in the CC [5] related to vascular differentiation and endothelial activation after Ebola virus infection. In our study, Tie2 is slightly elevated at day 7 in an asymptomatic line with detectable WNV in the brain.

Together, these findings build upon previous studies [5] where a genetic resource population (GRP) like the CC can detect key genes that may be driving the outcome variation in a population. In summary, the CC is emerging as a powerful resource for studying genetic variances that determine disease susceptibility, and utilizing sensitive, high throughput technologies, like nanostring to evaluate gene expression in GRPs will help us improve our understanding of host responses to pathogenic infection.

## Figures and Tables

**Fig. 1:**
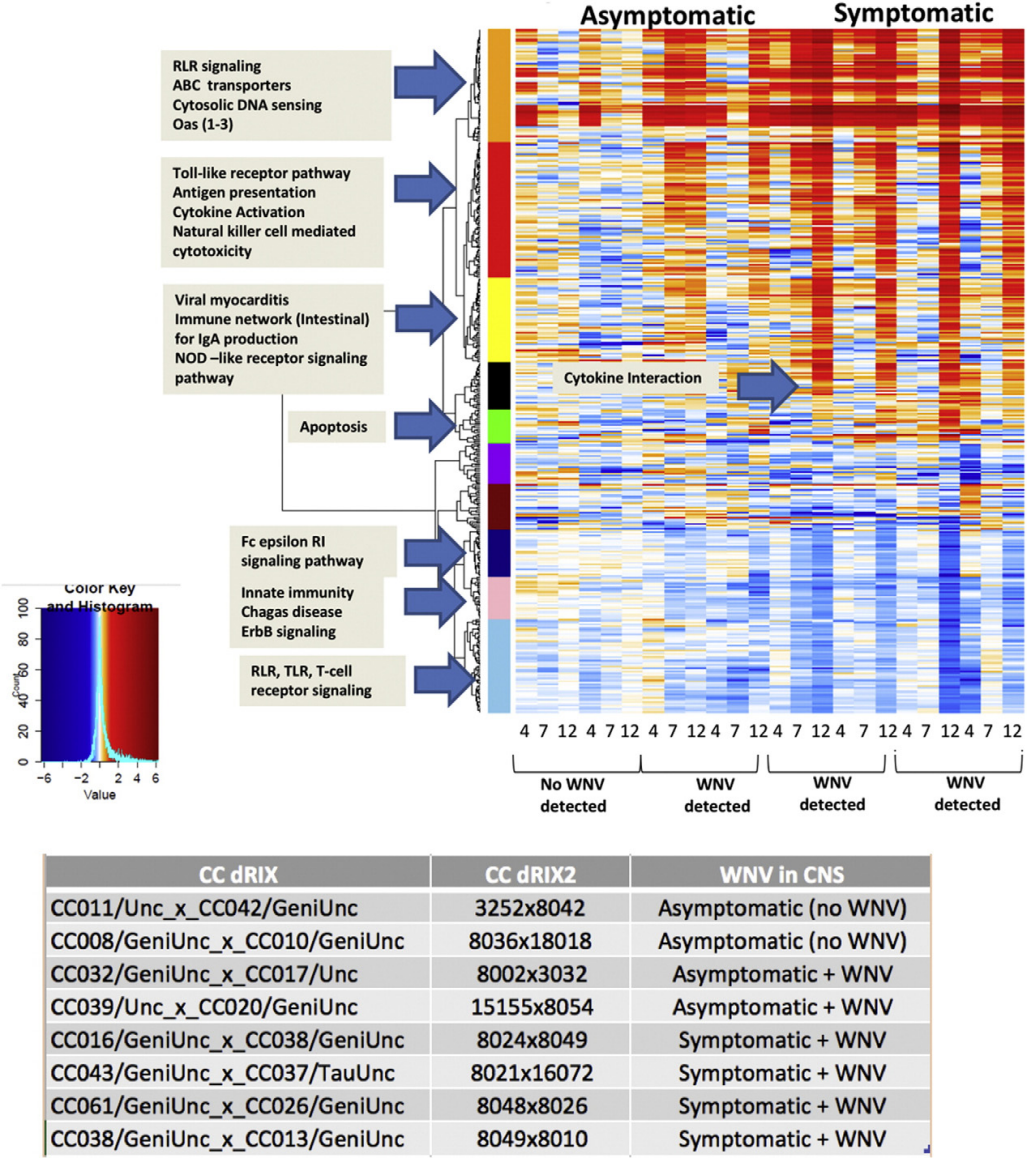
Genomic analysis in Collaborative Cross brain tissue. A heat map showing gene co-expression data across the CC-dRIX panel (Fig. 1). RNA was extracted from tissue isolated at days 4, 7, and 12 post-WNV infection, processed for nanostring, and differential expression assessed (relative to mock day 2). Table 1 shows the different genetic backgrounds of the CC-dRIX and their outcomes after exposure to WNV. CC dRIXs are sorted by disease pathogenic phenotype (asymptomatic dRIXs displayed no significant increase in clinical scores or weight loss, and fully recovered; symptomatic CC dRxs displayed increase in clinical scores, weight loss, and in some cases succumbed to infection). Genes are clustered by co-expressed modules (color blocks, Y-axis); identities in Table 2, see 3.7 co-expression).

**Table 1:** Summary of collaborative cross samples.

CC dRIX	WNV in CNS	Tissue	Timepoints
CC11/Unc_x_CC042/GeniUNc	Asymptomatic (no WNV)	Brain	D4, D7, D12
CC008/Geni_x_CC010/GeniUnc	Asymptomatic (no WNV)	Brain	D4, D7, D12
CC031/GeniUnc_x_CC017/Unc	Asymptomatic + WNV	Brain	D4, D7, D12
CC039/Unc_x_CC020/GeniUnc	Asymptomatic + WNV	Brain	D4, D7, D12
CC016/GeniUnc_x_CC038/GeniUnc	Symptomatic + WNV	Brain	D4, D7, D12
CC043/GeniUnc_x_CC037/TauUnc	Symptomatic + WNV	Brain	D4, D7, D12
CC061/GeniUnc_x_CC026/GeniUnc	Symptomatic + WNV	Brain	D4, D7, D12
CC038/GeniUnc_x_CC013/GeniUnc	Symptomatic + WNV	Brain	D4, D7, D12

**Table 2:** Identifiers and phenotypes for CC dRIXs (collaborative cross discovery recombinant intercrosses).

CC dRIX	CC dRIX2	WNV in CNS
CC11/Unc_x_CC042/GeniUNc	3252 × 8042	Asymptomatic (no WNV)
CC008/Geni_x_CC010/GeniUnc	8036 × 18018	Asymptomatic (no WNV)
CC031/GeniUnc_x_CC017/Unc	8002 × 3032	Asymptomatic + WNV
CC039/Unc_x_CC020/GeniUnc	15155 × 8054	Asymptomatic + WNV
CC016/GeniUnc_x_CC038/GeniUnc	8024 × 8049	Symptomatic + WNV
CC043/GeniUnc_x_CC037/TauUnc	8021 × 16072	Symptomatic + WNV
CC061/GeniUnc_x_CC026/GeniUnc	8048 × 8026	Symptomatic + WNV
CC038/GeniUnc_x_CC013/GeniUnc	8049 × 8010	Symptomatic + WNV
